# Sex-specific cortical, hippocampal and thalamic whole genome transcriptome data from controls and a G72 schizophrenia mouse model

**DOI:** 10.1186/s13104-024-06799-4

**Published:** 2024-05-21

**Authors:** Anna Papazoglou, Christina Henseler, Sandra Weickhardt, Johanna Daubner, Teresa Schiffer, Karl Broich, Jürgen Hescheler, Agapios Sachinidis, Dan Ehninger, Britta Haenisch, Marco Weiergräber

**Affiliations:** 1 Experimental Neuropsychopharmacology, Federal Institute for Drugs and Medical Devices (Bundesinstitut für Arzneimittel und Medizinprodukte, BfArM), Kurt Georg-Kiesinger-Allee 3, Bonn, 53175 Germany; 2 Federal Institute for Drugs and Medical Devices (Bundesinstitut für Arzneimittel und Medizinprodukte BfArM), Kurt-Georg-Kiesinger-Allee 3, Bonn, 53175 Germany; 3 Faculty of Medicine, Institute of Neurophysiology, University of Cologne, Robert-Koch-Str. 39, Cologne, 50931 Germany; 4 Center of Physiology and Pathophysiology, Faculty of Medicine, University of Cologne, Robert-Koch-Str. 39, Cologne, 50931 Germany; 5 Translational Biogerontology, German Center for Neurodegenerative Diseases (Deutsches Zentrum für Neurodegenerative Erkrankungen, DZNE), Venusberg-Campus 1/99, Bonn, 53127 Germany; 6 German Center for Neurodegenerative Diseases (Deutsches Zentrum für Neurodegenerative Erkrankungen, DZNE), Venusberg-Campus 1/99, Bonn, 53127 Germany; 7 Center for Translational Medicine, Medical Faculty, University of Bonn, Bonn, 53113 Germany

**Keywords:** Brain, Fold change, Hippocampus, Hybridization, Microarray, Retrosplenial cortex, RNA, Schizophrenia, Thalamus, Transcriptome

## Abstract

**Objectives:**

The G72 mouse model of schizophrenia represents a well-known model that was generated to meet the main translational criteria of isomorphism, homology and predictability of schizophrenia to a maximum extent. In order to get a more detailed view of the complex etiopathogenesis of schizophrenia, whole genome transcriptome studies turn out to be indispensable. Here we carried out microarray data collection based on RNA extracted from the retrosplenial cortex, hippocampus and thalamus of G72 transgenic and wild-type control mice. Experimental animals were age-matched and importantly, both sexes were considered separately.

**Data description:**

The isolated RNA from all three brain regions was purified, quantified und quality controlled before initiation of the hybridization procedure with SurePrint G3 Mouse Gene Expression v2 8  ×  60 K microarrays. Following immunofluorescent measurement und preprocessing of image data, raw transcriptome data from G72 mice and control animals were extracted and uploaded in a public database. Our data allow insight into significant alterations in gene transcript levels in G72 mice and enable the reader/user to perform further complex analyses to identify potential age-, sex- and brain-region-specific alterations in transcription profiles and related pathways. The latter could facilitate biomarker identification and drug research and development in schizophrenia research.

## Objective

G72/G30 is a primate specific gene that is localized on human chromosome 13q and turned out to be a susceptibility locus for major psychiatric disorders such as schizophrenia and bipolar disorder [1–3]. The dominant gene product of this locus is the largest G72 splice variant, i.e., LG72 protein (here referred to as G72 protein). Notably, the function of the G72 protein in neuropsychiatry remains obscure [4]. Interestingly, increased G72 protein levels were observed in the serum and CNS of schizophrenic patients [5, 6]. In order to investigate the functional implications of the G72 protein in vivo, a humanized transgenic mouse model carrying the G72/G30 locus has been generated that expresses the G72 protein [7]. G72 transgenic mice serve as a schizophrenia model as they display characteristic symptoms, such as motor coordination deficits, altered sensorimotor gating and olfactory discrimination, increased compulsive behavior and spatial memory impairment [7, 8].

Previous complementary proteomics studies from the cerebellum of G72 mice revealed altered protein expression in mitochondria-associated, myelin- and oxidative stress-related processes in G72 compared to control mice [8–11]. Recently, Filiou et al. (2022) performed multi-omics approaches, i.e., quantitative proteomics and metabolomics, from the hippocampus of male, eight weeks old G72 vs. control mice [10]. However, we are still lacking detailed information about both sex- and brain-region specific differences in transcriptome profiles from schizophrenia mouse models such as G72. To fill this essential gap, we carried out microarray analysis of RNA from the retrosplenial (RS) cortex, hippocampus and thalamus of both female and male G72 mice. It has been shown in the past that the RS cortex is essential for gating information to the medial temporal lobe and displays aberrant connectivity with neuronal networks associated with memory formation and ecphoria, i.e., the limbic system/hippocampal formation in schizophrenia patients [12]. In addition, enhanced neuronal connectivity of the RS cortex with the left superior temporal gyrus was observed in patients with more positive symptoms, e.g. hallucinations [12]. Impaired function of the RS cortex was also related to verbal memory deficits commonly seen in schizophrenia patients [13]. These and other findings suggest that the RS cortex is of central importance in schizophrenia symptomatology [12, 14]. The hippocampus as a memory consolidation and formation system has been related to schizophrenia for long. The etiopathological evolution of schizophrenia via the premorbid, prodromal to syndromal psychotic stages was shown to initiate with dysregulation of glutamate transmission in the CA1 area, further progressing to other hippocampal regions and cortical areas as well [15, 16]. The thalamus as part of the thalamocortical-corticothalamic circuitry serves as another important structure in schizophrenia. It’s critical for the transmission and processing of external information and thereby modulates essential tasks such as wakefulness, sleep and memory. It was shown in both animal models and humans that there are thalamic connectivity deficits in psychiatric disorders, including schizophrenia [17]. There is also evidence of altered thalamic microstructure, e.g., in the mediodorsal nucleus, thalamo-prefrontal and thalamo-somatosensory/parietal connectivity [17]. Multiple studies have proposed an association between psychosis spectrum disorders and thalamic network dysrhythmia and/or dysconnectivity [18, 19]. Our microarray data from these three highly relevant brain regions allow for further characterization of differentially expressed intersectional and signature genes of the individual subgroups, gene ontology and pathway analysis and biophysical studies [20, 21] which are of relevance for future translational studies.

## Data description

### Experimental animals

G72 transgenic mice carrying the G72/G30 locus and wild-type (WT) mice with a CD1 background were kindly provided by the Institute of Molecular Psychiatry (Life & Brain, Bonn, Germany). Details on breeding and genetics of G72 mice have been described in detail before [7, 8]. In total, eight WT control animals (four ♂, age: 23.14 ± 0.00 wks; four ♀, age: 23.46 ± 0.11 wks) and eight G72 transgenic mice (four ♂, age: 23.14 ± 0.00 wks; four ♀, age: 23.14 ± 0.00 wks) were used for dissection of hippocampus, RS cortex and thalamus for subsequent transcriptome analysis.

### Genotyping - DNA preparation from tail biopsies

Every experimental animal was genotyped twice using DNA isolated from tail biopsies. DNA preparation was carried out using peqGOLD DNA Mini Kit (PEQLAB Biotechnologie GmbH, Germany) according to the manufacturer’s instructions. The isolated genomic DNA was stored at + 4 °C until further use.

### Hippocampus, RS cortex and thalamus preparation and tissue storage

Experimental animals were anaesthetized using i.p. injection of ketamine (100 mg/kg) / xylazine (10 mg/kg) and immediately decapitated. The brain was removed and placed in a clean RNase-free petri-dish filled with pure RNAlater reagent (Qiagen GmbH, Germany). The RS cortex, hippocampus and thalamus were bluntly dissected and immediately placed in 2 ml RNase free reaction tubes, snap frozen in liquid nitrogen and stored at -80 °C until RNA preparation. This instantaneous and fast processing was performed to eliminate potential effects of anaesthesia on early gene regulation.

### Retrosplenial cortex, hippocampal and thalamic RNA isolation

Total RNA from the individual brain regions was extracted using RNeasy Lipid Tissue Mini Kit (Qiagen GmbH, Germany) according to the manufacturer’s protocol. Quantity and quality of the RNA was evaluated using NanoDrop ND-1000 (ThermoFisher Scientific Inc., USA).

### Acquisition of transcriptome data and raw data extraction

Transcriptome data were acquired using the One-Color Microarray-Based Gene Expression system (Agilent Technologies Germany GmbH & Co. KG, Germany). In specific, the SurePrint G3 Mouse Gene Expression v2 8 × 60 K Microarray Kit (Agilent Technologies Germany GmbH & Co. KG, Germany) was used for RS cortex, hippocampal and thalamic tissue. All procedures were carried out according to the manufacturer’s instructions.

Raw data are based on fluorescence scanning using the Agilent SureScan Microarray Scanner and raw microarray image file processing using the Feature Extraction Software (both from Agilent Technologies Germany GmbH & Co. KG, Germany). Using GeneSpring Software (Agilent Technologies Germany GmbH & Co. KG, Germany), all information about probe names, fold changes, etc. were extracted and exported into txt.- and csv.-files to allow usage in other transcriptome analysis software in case GeneSpring software is not used and/or not available. These raw data represent unfiltered, non pre-analyzed data.

The raw data (date files 1–47) as well as the two MAGE-TAB format files (data files 48–49) were uploaded to the ArrayExpress repository and are freely accessible with the following accession ID: E-MTAB-13547. The reader might also use the related identifiers.org link, i.e., “https://identifiers.org/arrayexpress:E-MTAB-13547” [12].

The individual subgroups and related data are characterized in Table 1.

**Table 1:** Overview of data files/data sets (for quality control files of the individual data files see supplementary information)

Label	Name of data file/data set	File types (file extension)	Data repository and identifier (DOI or accession number)
Data file 1	Sample1_Female_G72_cortex_#1.txt	txt.-file	Sample1: ArrayExpress (Accession ID: E-MTAB-13547) [12]
Data file 2	Sample2_Female_G72_cortex_#2.txt	txt.-file	Sample2: ArrayExpress (Accession ID: E-MTAB-13547) [12]
Data file 3	Sample3_Female_G72_cortex_#3.txt	txt.-file	Sample3: ArrayExpress (Accession ID: E-MTAB-13547) [12]
Data file 4	Sample4_Female_G72_hippocampus_#1.txt	txt.-file	Sample4: ArrayExpress (Accession ID: E-MTAB-13547) [12]
Data file 5	Sample5_Female_G72_hippocampus_#2.txt	txt.-file	Sample5: ArrayExpress (Accession ID: E-MTAB-13547) [12]
Data file 6	Sample6_Female_G72_hippocampus_#3.txt	txt.-file	Sample6: ArrayExpress (Accession ID: E-MTAB-13547) [12]
Data file 7	Sample7_Female_G72_hippocampus_#4.txt	txt.-file	Sample7: ArrayExpress (Accession ID: E-MTAB-13547) [12]
Data file 8	Sample8_Female_G72_thalamus_#1.txt	txt.-file	Sample8: ArrayExpress (Accession ID: E-MTAB-13547) [12]
Data file 9	Sample9_Female_G72_thalamus_#2.txt	txt.-file	Sample9: ArrayExpress (Accession ID: E-MTAB-13547) [12]
Data file 10	Sample10_Female_G72_thalamus_#3.txt	txt.-file	Sample10: ArrayExpress (Accession ID: E-MTAB-13547) [12]
Data file 11	Sample11_Female_G72_thalamus_#4.txt	txt.-file	Sample11: ArrayExpress (Accession ID: E-MTAB-13547) [12]
Data file 12	Sample12_Female_WT_cortex_#1.txt	txt.-file	Sample12: ArrayExpress (Accession ID: E-MTAB-13547) [12]
Data file 13	Sample13_Female_WT_cortex_#2.txt	txt.-file	Sample13: ArrayExpress (Accession ID: E-MTAB-13547) [12]
Data file 14	Sample14_Female_WT_cortex_#3.txt	txt.-file	Sample14: ArrayExpress (Accession ID: E-MTAB-13547) [12]
Data file 15	Sample15_Female_WT_cortex_#4.txt	txt.-file	Sample15: ArrayExpress (Accession ID: E-MTAB-13547) [12]
Data file 16	Sample16_Female_WT_hippocampus_#1.txt	txt.-file	Sample16: ArrayExpress (Accession ID: E-MTAB-13547) [12]
Data file 17	Sample17_Female_WT_hippocampus_#2.txt	txt.-file	Sample17: ArrayExpress (Accession ID: E-MTAB-13547) [12]
Data file 18	Sample18_Female_WT_hippocampus_#3.txt	txt.-file	Sample18: ArrayExpress (Accession ID: E-MTAB-13547) [12]
Data file 19	Sample19_Female_WT_hippocampus_#4.txt	txt.-file	Sample19: ArrayExpress (Accession ID: E-MTAB-13547) [12]
Data file 20	Sample20_Female_WT_thalamus_#1.txt	txt.-file	Sample20: ArrayExpress (Accession ID: E-MTAB-13547) [12]
Data file 21	Sample21_Female_WT_thalamus_#2.txt	txt.-file	Sample21: ArrayExpress (Accession ID: E-MTAB-13547) [12]
Data file 22	Sample22_Female_WT_thalamus_#3.txt	txt.-file	Sample22: ArrayExpress (Accession ID: E-MTAB-13547) [12]
Data file 23	Sample23_Female_WT_thalamus_#4.txt	txt.-file	Sample23: ArrayExpress (Accession ID: E-MTAB-13547) [12]
Data file 24	Sample24_Male_G72_cortex_#1.txt	txt.-file	Sample24: ArrayExpress (Accession ID: E-MTAB-13547) [12]
Data file 25	Sample25_Male_G72_cortex_#2.txt	txt.-file	Sample25: ArrayExpress (Accession ID: E-MTAB-13547) [12]
Data file 26	Sample26_Male_G72_cortex_#3.txt	txt.-file	Sample26: ArrayExpress (Accession ID: E-MTAB-13547) [12]
Data file 27	Sample27_Male_G72_cortex_#4.txt	txt.-file	Sample27: ArrayExpress (Accession ID: E-MTAB-13547) [12]
Data file 28	Sample28_Male_G72_hippocampus_#1.txt	txt.-file	Sample28: ArrayExpress (Accession ID: E-MTAB-13547) [12]
Data file 29	Sample29_Male_G72_hippocampus_#2.txt	txt.-file	Sample29: ArrayExpress (Accession ID: E-MTAB-13547) [12]
Data file 30	Sample30_Male_G72_hippocampus_#3.txt	txt.-file	Sample30: ArrayExpress (Accession ID: E-MTAB-13547) [12]
Data file 31	Sample31_Male_G72_hippocampus_#4.txt	txt.-file	Sample31: ArrayExpress (Accession ID: E-MTAB-13547) [12]
Data file 32	Sample32_Male_G72_thalamus_#1.txt	txt.-file	Sample32: ArrayExpress (Accession ID: E-MTAB-13547) [12]
Data file 33	Sample33_Male_G72_thalamus_#2.txt	txt.-file	Sample33: ArrayExpress (Accession ID: E-MTAB-13547) [12]
Data file 34	Sample34_Male_G72_thalamus_#3.txt	txt.-file	Sample34: ArrayExpress (Accession ID: E-MTAB-13547) [12]
Data file 35	Sample35_Male_G72_thalamus_#4.txt	txt.-file	Sample35: ArrayExpress (Accession ID: E-MTAB-13547) [12]
Data file 36	Sample36_Male_WT_cortex_#1.txt	txt.-file	Sample36: ArrayExpress (Accession ID: E-MTAB-13547) [12]
Data file 37	Sample37_Male_WT_cortex_#2.txt	txt.-file	Sample37: ArrayExpress (Accession ID: E-MTAB-13547) [12]
Data file 38	Sample38_Male_WT_cortex_#3.txt	txt.-file	Sample38: ArrayExpress (Accession ID: E-MTAB-13547) [12]
Data file 39	Sample39_Male_WT_cortex_#4.txt	txt.-file	Sample39: ArrayExpress (Accession ID: E-MTAB-13547) [12]
Data file 40	Sample40_Male_WT_hippocampus_#1.txt	txt.-file	Sample40: ArrayExpress (Accession ID: E-MTAB-13547) [12]
Data file 41	Sample41_Male_WT_hippocampus_#2.txt	txt.-file	Sample41: ArrayExpress (Accession ID: E-MTAB-13547) [12]
Data file 42	Sample42_Male_WT_hippocampus_#3.txt	txt.-file	Sample42: ArrayExpress (Accession ID: E-MTAB-13547) [12]
Data file 43	Sample43_Male_WT_hippocampus_#4.txt	txt.-file	Sample43: ArrayExpress (Accession ID: E-MTAB-13547) [12]
Data file 44	Sample44_Male_WT_thalamus_#1.txt	txt.-file	Sample44: ArrayExpress (Accession ID: E-MTAB-13547) [12]
Data file 45	Sample45_Male_WT_thalamus_#2.txt	txt.-file	Sample45: ArrayExpress (Accession ID: E-MTAB-13547) [12]
Data file 46	Sample46_Male_WT_thalamus_#3.txt	txt.-file	Sample46: ArrayExpress (Accession ID: E-MTAB-13547) [12]
Data file 47	Sample47_Male_WT_thalamus_#4.txt	txt.-file	Sample47: ArrayExpress (Accession ID: E-MTAB-13547) [12]
Data file 48	E-MTAB-13547.idf.txt (Metadata in the MAGE-TAB format: investigation description format file)	txt.-file	ArrayExpress (Accession ID: E-MTAB-13547) [12]
Data file 49	E-MTAB-13547.sdrf.txt (Metadata in the MAGE-TAB format: sample data relationship format file)	txt.-file	ArrayExpress (Accession ID: E-MTAB-13547) [12]

## Limitations

This transcriptome data collection from the RS cortex, hippocampus and thalamus of ∼ 6 months old G72 and control mice was carried out using a microarray approach. Following scientific and organizational aspects, resource considerations, and power calculations, this turned out to be a feasible approach for us. We are aware that RNAseq is also a very good alternative strategy to characterize transcriptional alterations in the G72 schizophrenia mouse line.

The G72 mouse model is a unique schizophrenia model to characterize the impact of the G72 protein in neuropsychiatry [7, 8]. This may limit its generalizability compared to other schizophrenia models as regards isomorphism, homology and predictability representing the major translational categories in biomedicine. As for other diseases, numerous schizophrenia mouse models have been generated and none of these models can claim to mimic the etiopathogenesis, symptomatology and drug delivery characteristics of the human disease equivalent to a 100% [22–24]. Given these observations, searching a schizophrenia model with common trans-line and trans-species transcriptional profiles is highly challenging. We have thus decided to use the humanized transgenic mouse model G72 carrying the G72/G30 locus which has received much attention in literature, and which can provide novel insight into the crucial role of G72 protein in the etiopathogenesis of schizophrenia.

A unique feature of our data is related to their sex- and brain region-related specificity. Clearly, this results in a high number of experimental subgroups (twelve in total) and microarrays to be used. Note that we focused on animals of “older” age (i.e., 23 wks) in this data collection as stereotypic behavior, characterized by repetitive and unintentional movements typically appears in progressed disease stages [25–28]. To get an impression of temporal effects on G72 transcriptome profiles, additional studies with G72 and control animals of different ages are necessary.

## Data Availability

The data described in this Data note (data files 1–47 in total; files #1–3: female G72 cortex (*n* = 3); files #4–7: female G72 hippocampus (*n* = 4); files #8–11: female G72 thalamus (*n* = 4); files # 12–15: female WT cortex (*n* = 4); files #16–19: female WT hippocampus (*n* = 4); files #20–23: female WT thalamus (*n* = 4); files #24–27: male G72 cortex (*n* = 4); files #28–31: male G72 hippocampus (*n* = 4); files #32–35: male G72 thalamus (*n* = 4); files #36–39: male WT cortex (*n* = 4); files #40–43: male WT hippocampus (*n* = 4); files #44–47: male WT thalamus (*n* = 4); and the files 48 and 49 (metadata in the MAGE-TAB format) can be freely and openly accessed in the ArrayExpress repository using the following accession ID: E-MTAB-13547. The reader might also use the related identifiers.org link, i.e., “https://identifiers.org/arrayexpress: E-MTAB-13547” [12].

## Abbreviations

RS: Retrosplenial
WT: Wild-type

## References

[1] Chumakov I, Blumenfeld M, Guerassimenko O, Cavarec L, Palicio M, Abderrahim H, Bougueleret L, Barry C, Tanaka H, La Rosa P (2002). Genetic and physiological data implicating the new human gene G72 and the gene for D-amino acid oxidase in schizophrenia. Proc Natl Acad Sci U S A.

[2] Detera-Wadleigh SD, McMahon FJ (2006). G72/G30 in schizophrenia and bipolar disorder: review and meta-analysis. Biol Psychiatry.

[3] Jansen A, Krach S, Krug A, Markov V, Eggermann T, Zerres K, Thimm M, Nothen MM, Treutlein J, Rietschel M (2009). Effect of the G72 (DAOA) putative risk haplotype on cognitive functions in healthy subjects. BMC Psychiatry.

[4] Sacchi S, Binelli G, Pollegioni L (2016). G72 primate-specific gene: a still enigmatic element in psychiatric disorders. Cell Mol Life Sci.

[5] Akyol ES, Albayrak Y, Aksoy N, Sahin B, Beyazyuz M, Kuloglu M, Hashimoto K (2017). Increased serum G72 protein levels in patients with schizophrenia: a potential candidate biomarker. Acta Neuropsychiatr.

[6] Korostishevsky M, Kaganovich M, Cholostoy A, Ashkenazi M, Ratner Y, Dahary D, Bernstein J, Bening-Abu-Shach U, Ben-Asher E, Lancet D (2004). Is the G72/G30 locus associated with schizophrenia? Single nucleotide polymorphisms, haplotypes, and gene expression analysis. Biol Psychiatry.

[7] Otte DM, Bilkei-Gorzo A, Filiou MD, Turck CW, Yilmaz O, Holst MI, Schilling K, Abou-Jamra R, Schumacher J, Benzel I (2009). Behavioral changes in G72/G30 transgenic mice. Eur Neuropsychopharmacol.

[8] Otte DM, Sommersberg B, Kudin A, Guerrero C, Albayram O, Filiou MD, Frisch P, Yilmaz O, Drews E, Turck CW (2011). N-acetyl cysteine treatment rescues cognitive deficits induced by mitochondrial dysfunction in G72/G30 transgenic mice. Neuropsychopharmacology.

[9] Filiou MD, Martins-de-Souza D, Guest PC, Bahn S, Turck CW (2012). To label or not to label: applications of quantitative proteomics in neuroscience research. Proteomics.

[10] Filiou MD, Teplytska L, Nussbaumer M, Otte DM, Zimmer A, Turck CW. Multi-omics Analysis reveals myelin, presynaptic and nicotinate alterations in the Hippocampus of G72/G30 transgenic mice. J Pers Med 2022, 12(2). 10.3390/jpm12020244 PMC8878587 35207732

[11] Filiou MD, Turck CW (2012). Psychiatric Disorder biomarker discovery using quantitative proteomics. Methods Mol Biol.

[12] Bluhm RL, Miller J, Lanius RA, Osuch EA, Boksman K, Neufeld RW, Theberge J, Schaefer B, Williamson PC (2009). Retrosplenial cortex connectivity in schizophrenia. Psychiatry Res.

[13] Tendolkar I, Weis S, Guddat O, Fernandez G, Brockhaus-Dumke A, Specht K, Klosterkotter J, Reul J, Ruhrmann S (2004). Evidence for a dysfunctional retrosplenial cortex in patients with schizophrenia: a functional magnetic resonance imaging study with a semantic-perceptual contrast. Neurosci Lett.

[14] Rolls ET (2021). Attractor cortical neurodynamics, schizophrenia, and depression. Transl Psychiatry.

[15] Lieberman JA, Girgis RR, Brucato G, Moore H, Provenzano F, Kegeles L, Javitt D, Kantrowitz J, Wall MM, Corcoran CM (2018). Hippocampal dysfunction in the pathophysiology of schizophrenia: a selective review and hypothesis for early detection and intervention. Mol Psychiatry.

[16] Wegrzyn D, Juckel G, Faissner A. Structural and functional deviations of the Hippocampus in Schizophrenia and Schizophrenia Animal models. Int J Mol Sci 2022, 23(10). 10.3390/ijms23105482 PMC9143100 35628292

[17] Hwang WJ, Kwak YB, Cho KIK, Lee TY, Oh H, Ha M, Kim M, Kwon JS (2022). Thalamic connectivity System Across Psychiatric disorders: current status and clinical implications. Biol Psychiatry Glob Open Sci.

[18] Anticevic A, Halassa MM (2023). The thalamus in psychosis spectrum disorder. Front Neurosci.

[19] Angulo Salavarria MM, Dell’Amico C, D’Agostino A, Conti L, Onorati M (2023). Cortico-thalamic development and disease: from cells, to circuits, to schizophrenia. Front Neuroanat.

[20] Panda D, Saha P, Chaudhuri R, Prasanth T, Ravichandiran V, Dash J (2019). A competitive pull-down assay using G-quadruplex DNA linked magnetic nanoparticles to determine specificity of G-quadruplex ligands. Anal Chem.

[21] Chaudhuri R, Prasanth T, Dash J (2023). Expanding the Toolbox of Target Directed Bio-orthogonal Synthesis: in situ direct macrocyclization by DNA templates. Angew Chem Int Ed Engl.

[22] Jones CA, Watson DJ, Fone KC (2011). Animal models of schizophrenia. Br J Pharmacol.

[23] Winship IR, Dursun SM, Baker GB, Balista PA, Kandratavicius L, Maia-de-Oliveira JP, Hallak J, Howland JG (2019). An overview of animal models related to Schizophrenia. Can J Psychiatry.

[24] Bialon M, Wasik A. Advantages and limitations of Animal Schizophrenia models. Int J Mol Sci 2022, 23(11). 10.3390/ijms23115968 PMC9181262 35682647

[25] Ridley RM (1994). The psychology of perserverative and stereotyped behaviour. Prog Neurobiol.

[26] Cheng L, Hattori E, Nakajima A, Woehrle NS, Opal MD, Zhang C, Grennan K, Dulawa SC, Tang YP, Gershon ES (2014). Expression of the G72/G30 gene in transgenic mice induces behavioral changes. Mol Psychiatry.

[27] Morrens M, Hulstijn W, Lewi PJ, De Hert M, Sabbe BG (2006). Stereotypy in schizophrenia. Schizophr Res.

[28] Garner JP, Meehan CL, Mench JA (2003). Stereotypies in caged parrots, schizophrenia and autism: evidence for a common mechanism. Behav Brain Res.

